# Posterior reversible encephalopathy syndrome in the setting of trauma: A case report

**DOI:** 10.1016/j.ijscr.2020.06.061

**Published:** 2020-06-18

**Authors:** Joshua Braganza, Abimbola Pratt

**Affiliations:** Department of Surgery, Hackensack Meridian Jersey Shore University Medical Center, United States

**Keywords:** Posterior reversible encephalopathy syndrome (PRES), Intracranial hypertension, Traumatic brain injury, Neuroimaging, Trauma

## Abstract

Reports of posterior reversible encephalopathy syndrome (PRES) in the setting of trauma and acute care surgery are scarce. PRES presents rapidly with a variety of symptoms including headaches, visual disturbances, altered consciousness, and seizures. It is associated with acute hypertensive episodes. PRES is diagnosed with a specific neuroimaging pattern and a constellation of clinical symptoms. This case report presents two traumatically injured patients with one confirmed case of PRES and the other with a potential case of PRES. The diagnosis was made through neuroimaging showing patchy T2 and diffusion hyperintensity in the periphery of both occipital lobes and adjacent cerebellar hemispheres on MRI in one case. The other case highlights extensive stable white matter disease without evidence of acute infarct on MRI, as well as diminished attenuation within the cerebral white matter in the occipital lobes on CT scan. There was resolution of visual symptoms in one patient while the other patient's neurologic status did not allow for evaluation of symptom resolution. This report aims to emphasize the possibility of PRES in trauma patients with a specific pattern of neuroimaging and clinical symptoms, and to increase the index of suspicion in acute care providers.

## Highlights

• Posterior reversible encephalopathy syndrome (PRES) in the setting of trauma and acute care surgery.
• Posterior reversible encephalopathy syndrome (PRES) in trauma patients with a specific pattern of neuroimaging and clinical symptoms.
• T2 weighted diffusion hyperintensities were present on MRI of the brain in both cases.

## 1 Introduction

There are few reports of posterior reversible encephalopathy syndrome (PRES) in the setting of trauma and acute care surgery. PRES presents rapidly with symptoms including headaches, visual disturbances, altered consciousness, and seizures. It is associated with acute hypertensive episodes. The etiology and pathophysiology of PRES is unclear, however there are two proposed mechanisms. The first involves a failed autoregulation in the setting of severe hypertension, resulting in endothelial injury and vasogenic edema [7]. The second proposes that vasoconstriction and hypoperfusion leads to ischemic cytotoxicity and subsequent vasogenic edema [6]. PRES is diagnosed with a specific neuroimaging pattern and a constellation of clinical symptoms. Herein, we present two traumatically injured patients with one confirmed case of PRES and another with a potential case of PRES. Ultimately, this report aims to emphasize the possibility of PRES in trauma patients, with a specific pattern of neuroimaging and clinical symptoms, with the goal being to increase the index of suspicion in acute care providers. This case report has been reported in line with SCARE 2018 criteria [1].

## 2 Case report

### 2.1 Case 1

A 40-year-old woman (height:5′0″ and weight: 125lbs) was brought to our Level II trauma center following a motor vehicle collision in mid-May of 2018. She was a restrained driver in the crash and denied losing consciousness. Her Glasgow coma scale (GCS) was 15. She reported abdominal pain and had abrasions over her left clavicle and lower abdomen in a seatbelt sign pattern, contusions over her right thigh and left hip and a laceration over her right knee revealing the joint. CT scan of the head, chest, abdomen, and pelvis was performed, and small foci of air was seen under the diaphragm which was indicative for small bowel perforation. A CT Scan of the head without IV contrast revealed no acute processes, including intracranial hemorrhage or cerebral contusions. The patient was emergently taken to the operating room for an exploratory laparotomy and irrigation and debridement of the right lower extremity.

The patient’s prior past medical history was significant for hypothyroidism and medications prescribed were Synthroid 88 mg once daily. The patient reported that she was compliant with her medication. She denied any hypertension, cardiac, pulmonary, renal, neurological or substance use history. The patient had a normal sinus rhythm and a normal ECG with all intervals within normal limits.

On anesthesia pre-operative exam, the patient’s vital signs were within normal limits. The patient underwent an exploratory laparotomy for a perforation in the jejunum and a tear in the mesentery followed by an irrigation and debridement for the open laceration in the right knee. Intraoperatively, the patient’s vitals fluctuated. The patient’s blood pressure ranged from 80 to 140/ 38 to 69 mmHg. The heart rate ranged from 79 to 125 beats per minute (bpm). The patient received 160mcg of phenylephrine at induction,and received 10 mg of ephedrine and 240mcg of phenylephrine when her blood pressure dropped to 90/39 mmHg.

In recovery, the patient’s vital signs were as follows: blood pressure of 93/71 mmHg, heart rate of 104 beats per minute, respiratory rate of 19 breaths per minute, and an O_2_ saturation of 96% on room air. The patient’s vital signs were monitored continuously in the post anesthesia care unit. On discharge from the recovery room, her vitals were as follows: blood pressure of 136/69 mmHg, 98 bpm, 18 breaths per minute with a 96% O_2_ saturation on room air.

The patient’s hospital course was prolonged due to a tarsometatarsal fracture which required an open reduction internal fixation. Preoperatively, the patient’s vitals were as follows: blood pressure of 116/58 mmHg, heart rate of 101 bpm, 16 breaths per minute and saturating 94% on room air. Intraoperatively, the patient’s vital signs fluctuated, with her blood pressure ranging from 85 to 150/41 to 78 mmHg. Her heart rate ranged from 60 to 130 bpm. The patient received 80mcg of phenylephrine when her blood pressure dropped to 85/42 mmHg.

In recovery, the patient’s vital signs were as follows: blood pressure of 135/77 mmHg, heart rate of 95 beats per minute, respiratory rate of 24 breaths per minute, and an O_2_ saturation of 96% on room air. The patient’s vital signs were monitored continuously in the post anesthesia care unit. Her vitals were blood pressure of 123/74 mmHg, 89 beats per minute, 17 breaths per minute with a 96% O_2_ saturation on room air prior to discharge from the recovery room.

During her stay, her vital signs were within normal limits with the exception of episodes of sinus tachycardia as high as 119. The patient’s blood pressure was well controlled, and her systolic pressure never was higher than 140.

**Fig. 1:**
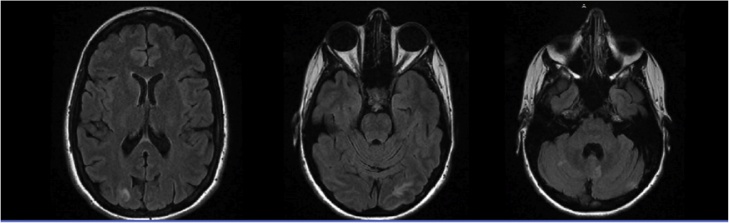
MRI imaging showing the patchy T2 and diffusion hyperintensity in the occipital loves and adjacent cerebellar hemisphere.

On post exploratory laparotomy day 3, the patient developed a headache and visual changes manifesting as central blurriness. Ophthalmology was consulted and attributed the cause of visual changes to macular edema. No intervention was recommended due to their belief that it would resolve on its own. The headache and visual disturbances persisted through day 7 and now progressed to right sided hemianopsia. An MRI of the brain, MRA of the head without contrast and MRA of the neck with contrast were ordered to rule out stroke and intracranial hemorrhage. The MRA of the head and neck showed no findings, however the MRI brain showed some patchy T2 areas and diffusion hyperintensity in the periphery of both occipital lobes and adjacent cerebellar hemispheres. These hyperintensities extended superiorly on the right side into the parieto-occipital junction while the left hyperintensity appeared to spread more inferiorly and laterally (*See* Fig. 1). These findings were consistent with posterior reversible encephalopathy syndrome.

Over the next 3 days, the patient received an open reduction internal fixation for the tarsometatarsal fracture and was discharged to rehabilitation. The patient reports that her hemianopsia resolved in mid-July of 2018.

### 2.2 Case 2

**Fig. 2:**
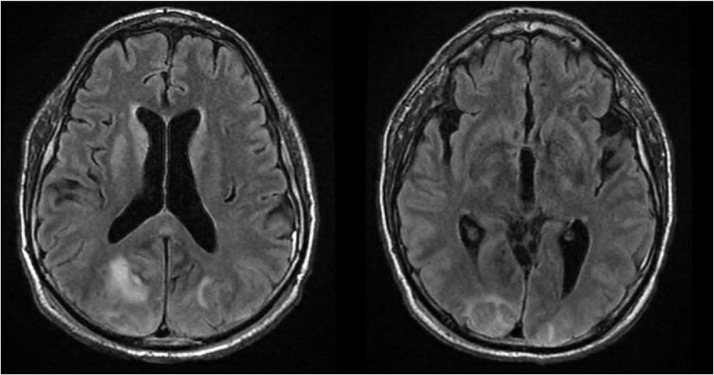
MRI showing area of ischemia in right occipital lobe possibly due to right vertebral artery dissection.

A 56-year-old man (height: 5′11″ and weight: 194lbs) was brought to the ED via ambulance after being assaulted by multiple individuals and run over by a motor vehicle. The patient was brought into our Level II trauma center as a trauma code and had CPR on entry into the trauma bay. The patient had suffered blunt trauma to the face with orbital ecchymoses and maxillary edema, traumatic brain injury (TBI) with no discrete hemorrhages but soft tissue emphysema of the anterior neck, extensive soft tissue swelling overriding the right face, Hydrocephalus, bilateral pneumothoraxes, bilateral pulmonary contusions, multiple rib fractures including right ribs 1 through 8 anteriorly; right ribs 4 through 6 laterally; left ribs 2 through 7 anteriorly; left ribs 3, 4, 5, 8, 9, 10, 11 laterally, flail chest, hypoxic and hypercapnic respiratory failure, grade I pancreatic laceration, left scapula fracture, right sacral fracture extending into right sacroiliac joint, right superior and inferior pubic rami fracture, unstable right knee, unstable left knee, left humeral fracture, hemorrhagic shock. He required emergent tracheostomy, and massive transfusion protocol. The patient was GCS 3 t. Bilateral chest tubes were placed for the bilateral pneumothoraxes. Vitals post return of spontaneous circulation were blood pressure of 90/56 mmHg and a heart rate of 89 beats per minute. Resuscitation continued until the patient’s vitals were within normal limits. Bilateral occipital hemorrhages were seen on CT of the head which required insertion of an external ventricular drain. The patient was also maintained on Keppra (levetiracetam) for seizure prophylaxis. As the patient started to be weaned off of IV sedation, the opening intracranial pressures were stable and the external ventricular drain was removed on hospital day 8. A state of permissive hypertension was maintained to ensure adequate perfusion to the brain post cerebrovascular accident with pressures ranging from 140 to 181/64 to 89 mmHg. On hospital day 12, a gastric feeding tube was placed bedside in the surgical ICU. On hospital day 14 the patient was stable enough to undergo an MRI. The MRI showed possible vertebral artery dissection and neurosurgery was consulted (*See* Fig. 2).

Per neurosurgery, it was unlikely to be a vertebral artery dissection due to the lack of specific vertebrobasilar disruption on the initial CTA head and neck. No further studies were recommended due to the patient’s injuries associated with trauma and hemodynamic instability causing potential stroke and/or embolus.

On hospital day 30, the patient developed acute acalculous, cholecystitis and a percutaneous cholecystostomy tube was placed on hospital day 31. The patient remained in a persistent vegetative state, which resulted in a prolonged hospital stay. He developed Serratia bacteremia and Vancomycin resistant Entercocci (VRE) in the urine.

**Fig. 3:**
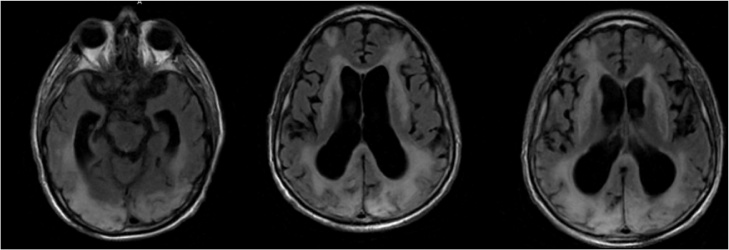
MRI showing extension stable white matter disease without evidence of acute infarct.

**Fig. 4:**
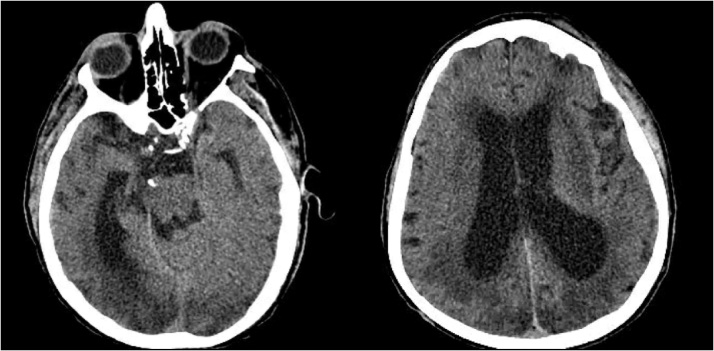
CT Scan of the head showing small linear areas of hyperintensity in the posterior occipito-parietal junction as well as enlarged ventricles suggesting the recurrence of hydrocephalus.

On hospital day 104, the patient underwent repeat MRI and a CT of the head due to his persistent vegetative state. The CT scan showed questionable small linear areas of hyperintensity in the posterior occipito-parietal junction as well as enlarged ventricles suggesting the recurrence of hydrocephalus. The MRI of the brain showed extensive stable white matter disease without evidence of acute infarct (*See* Fig. 3). A plan for a permanent ventriculoperitoneal (VP) shunt was made. On hospital day 127, another CT of the head was performed which revealed a diminished attenuation within the cerebral white matter (*See* Fig. 4). These findings along with those from hospital day 104, could have been suggestive of PRES, however, the neurologic status of the patient made it difficult to assess any clinical correlation. On hospital day 128, the patient had a VP shunt placed.

Over the patient’s 132-day long hospital course, the neurological status ranged from GCS 3 t–6 t with the only improvements observed being spontaneous opening of the eyes and brief periods of withdrawing to painful stimulus. The patient was eventually discharged to a long-term acute care facility on hospital day 132.

## 3 Discussion

Posterior Reversible Encephalopathy Syndrome (PRES) (also known as Reversible Posterior Leukoencephalopathy Syndrome) presents acutely with headaches, seizures, altered consciousness, visual disturbances including vision loss and cortical blindness. It is accompanied by abnormal findings on neuroimaging, characterized by symmetric bilateral subcortical/cortical hyperintensities in T2-weighted images, commonly in the occipito-parietal lobes [13]. PRES has also been described on CT as typically demonstrating focal regions of symmetric hemispheric edema [2,3,8]. This process was first identified in 1996 by Hinchey et al. and was termed RPLS [8]. This process was later proposed to be termed PRES by Casey et al. in 2000 to indicate the common involvement of grey and white matter involvement [5].

Although, the pathophysiology of PRES is not completely understood, it is thought to arise from one of two mechanisms. The first hypothesizes that a rapid rise in blood pressure may overcome the brain’s normal autoregulation of cerebral blood flow. This results in dilatation of cerebral arterioles with relaxation of endothelial tight junctions. Consequently, leakage of plasma and red blood cells into the extracellular space leads to cerebral edema. This process has a greater effect on cerebral white matter — given the presence of arterioles and capillaries within the cellular matrix of myelinated fiber tracts. These arterioles and capillaries make white matter more susceptible to vasogenic edema [7]. This hypothesis explains that PRES manifests in the occipito-parietal lobes due to a lack of sympathetic adrenergic innervation of the vertebrobasilar system. PRES occurs because the posterior cerebral blood vessels lack vasoconstrictor properties [9].

The second hypothesis states that vasospasm is the main driver of PRES, secondary to severe and sudden rises in blood pressure and ischemia of brain tissue. This hypothesizes that ischemia produces a cytotoxic edema and resulting in extracellular edema [6].

Several cases of PRES have been reported and associated with preeclampsia, eclampsia, hypertensive emergency, and general anesthesia complications. There have been few reports of PRES with traumatically injured patients. This report highlights a case of PRES in one trauma patient with a motor vehicle collision that resulted in a small bowel perforation and a tarsometatarsal fracture and another patient who was assaulted by multiple individuals and was run over by a motor vehicle.

In our first case, there were no hypertensive episodes during surgery or post-operatively. This establishes our case as a normotensive PRES case which has only been described in non-spinal surgery by Bartynski [2,3]. In another study by Berg et al., they report cortical blindness due to intraoperative hypotension. While this patient had post-operative hypotension, her symptoms of headache and right sided hemianopsia presented on day 7 where her blood pressure was still normotensive. Recent literature presents cases of PRES related to hypotension, yet all of these cases were related to spine surgery and spinal anesthesia [12]. There are some studies reporting PRES in patients with renal failure [4], hematological disorders [15], and transfusion [11] however, our patient had a normal basic metabolic panel (BMP) and CBC, and was not transfused.

In our second case, the neuroimaging and clinical correlation could not be made due to the neurological status of the patient. With respect to neuroimaging, a strong case for PRES could be made as there were hyperintensities in the occipito-parietal regions and extensive white matter disease in the parieto-occipital region. Whether this was residual from the bilateral occipital hemorrhages or from the permissive hypertension is yet to be determined. Given the patient’s persistent vegetative state, it is difficult to assess how this would manifest clinically. Seizures have frequently been linked to PRES. The patient was maintained on Keppra given his TBI and possible late post traumatic seizures (LPTS). Patients with LPTS have an increased mortality compared to patient’s who do not [10].

In both patients, it is possible that the edema in the occipito-parietal region may have been caused from traumatic brain injury, as both patients experienced TBI. Patients with TBI are often placed on vasopressors and require multiple transfusions for hypotensive episodes. Following administration of vasopressors and transfusions, the patients have a rapid correction of blood pressure, which could lead to edema specifically in the occipital lobes. In a case presented by Yoon, Lee, and Kim, a patient suffered severe head trauma and presented with PRES which resolved 3 months after initial presentation. Symptoms for this patient did not include nausea, headaches or visual disturbances, but rather seizures [14]. Given the broad symptomology for PRES, the variety of clinical ailments – many that are life and limb threatening – in trauma patients, the diagnosis of PRES may be overlooked by trauma teams.

## 4 Conclusion

There are few reports of PRES in the setting of trauma. This could be due to a low index of suspicion, uncertainty of etiology, or lack of awareness of the disease. It is important to consider PRES in trauma patients given great fluctuations in blood pressure, and associated TBI. The optimization of blood pressure fluctuations immediately following trauma may decrease the visual impairment, headaches, seizures, and the altered consciousness that define PRES.

## Declaration of Competing Interest

None.

## Funding

None.

## Ethical approval

This case report was exempt from ethical approval in our institution.

## Consent

Written informed consent was obtained from the patients or their families for publication of this case report and accompanying images. A copy of the written consent is available for review by the Editor-in-Chief of this journal on request.

## Author contribution

Joshua Braganza - Writing the paper, selecting the figures.

Abimbola Pratt MD - Surgeon in the cases, Case concept, Editor.

Contributors - Nicole L. Glynn MD Diagnostic Radiology.

## Registration of research studies

1. Name of the registry: Not Applicable.
2. Unique identifying number or registration ID: Not Applicable.
3. Hyperlink to your specific registration (must be publicly accessible and will be checked):

## Guarantor

Abimbola Pratt MD – Abimbola.pratt@hackensackmeridian.org.

## Learning objectives

To identify and manage posterior reversible encephalopathy syndrome (PRES) in traumatically injured patients.

## Provenance and peer review

Not commissioned, externally peer-reviewed.
